# Importance of Mean Red Cell Distribution Width in Hypertensive Patients

**DOI:** 10.7759/cureus.902

**Published:** 2016-11-29

**Authors:** Ahmed Bilal, Junaid H Farooq, Immad Kiani, Salman Assad, Haider Ghazanfar, Imran Ahmed

**Affiliations:** 1 Department of Internal Medicine, Military Hospital, Rawalpindi, Pakistan; 2 Yusra Medical and Dental College, Islamabad, Pakistan; 3 Internal Medicine, Shifa International Hospital, Islamabad, Pakistan; 4 Department of Medicine, Shifa Tameer-e-Millat University, Islamabad, Pakistan; 5 Department of Neurology, Shifa International Hospital, Islamabad, Pakistan

**Keywords:** hypertension, erythrocyte indices, inflammation

## Abstract

**Purpose:**

Red cell distribution width (RDW), expressed in femtoliters (fl), is a measure of the variation in the size of circulating erythrocytes and is often expressed as a direct measurement of the width of the distribution. We aim to observe the mean value of red cell distribution width (RDW) in hypertensive patients. Increased RDW can be used as a tool for early diagnosis, as an inflammatory marker, and a mortality indicator in hypertensive patients due to its close relation to inflammation.

**Materials and methodology:**

Hypertensive patients who had the condition for more than one year duration, diagnosed according to the Joint National Committee (JNC 7) criteria were subjected to complete blood count and RDW measurement. One hundred patients, aged between 12 years and 65 years were enrolled from the outpatient department of medicine at the Military Hospital Rawalpindi.

**Results:**

The mean age (± SD) of the patients was 51.48 ± 10.08 years. Out of 100 patients 69% were males whereas 31% were females. The overall frequency of hypertension more than five years was 55% subjects whereas 45% individuals had duration of hypertension less than five years. Mean RDW in females was found to be 49.35±8.42 fl while mean RDW in males was 44.78±7.11 fl. An independent sample t-test was applied to assess if there was any significant difference between age and gender. No significant difference between age and gender was found (p<0.05). The Mann-Whitney test was used to assess any association of RDW with gender. RDW values in females was found to be statistically significantly higher than in males (U=603, p=0.01). Linear regression showed that mean RDW value increased with increasing age (P <0.001).

**Conclusions:**

A significant number of patients with hypertension have increased levels of RDW. Therefore, it is recommended that serum RDW should be checked regularly in patients with hypertension.

## Introduction

Red cell distribution width (RDW), a component of routine complete blood count (CBC) is a measure of the variation in the size of circulating erythrocytes. It is routinely measured by automated hematology analyzers and is often expressed as a direct measurement of the width of the distribution, which gives a measure in femtoliters (fl) [1]. RDW is an index of the heterogeneity of erythrocytes (i.e. anisocytosis) [2]. Used in normal clinical setting as tool to differentiate between different types of anemia, recent studies have shown RDW as predictor of mortality in multiple clinical conditions [3-4]. RDW's association as a prognostic marker predicting a worse prognosis in several diseases such as coronary heart disease (CHD), stroke, peripheral artery disease (PAD), heart failure (HF), venous thromboembolism (VTE) and pulmonary arterial hypertension (PAH) is well documented [5-11].

Increased RDW is common in patients with deficiencies of iron, folate, and vitamin B12 but normal in thalassemia [12]. Macrocytosis is associated with increased RDW, but RDW is usually normal in macrocytosis [13]. Increased RDW is also seen in hemolytic anemia, transfusion reactions, beta thalassemia, and anemia of chronic disorders, hereditary spherocytosis, and sickle cell anemia [14]. It has also been associated with chronic hepatobiliary disease, hypothyroidism, Behçet’s disease, systemic lupus erythematosus and inflammatory bowel disease. RDW is also associated with increasing age, obesity, low cardio-respiratory fitness, smoking, being unmarried, and high alcohol consumption [15].

According to the 2013 European Society of Hypertension and European Society of Cardiology guidelines, the presence of subclinical organ damage is a fundamental factor in determining the estimated cardiovascular risk with proposed scale. Renal function damage is one of the common subclinical organ damages caused by hypertension [16]. Renal dysfunction is associated with poor cardiovascular outcome. Despite extensive research over the past several decades, the etiology of most cases of adult hypertension is still unknown, and control of blood pressure is suboptimal in the general population. Due to the associated morbidity and mortality and cost to society, preventing and treating hypertension is an important public health challenge. Fortunately, recent advances and trials in hypertension research are leading to an increased understanding of the pathophysiology of hypertension and the promise for novel pharmacologic and interventional treatments for this widespread disease [17-18].

Patients with hypertension and prehypertension have elevated RDW [4]. Approximately 90% of hypertension cases are classified as essential hypertension, where the precise cause is unknown. Hypertension is associated with inflammation; however, whether inflammation is a cause or effect of hypertension is not well understood [19]. The purpose of this study is to determine the role of RDW monitoring in the management of hypertension. Raised values of RDW will suggest the role of inflammation in the etiology of hypertension. Hence, monitoring of this inflammatory marker may be of value in the prediction of complications of hypertension. This research will provide us with a baseline for future studies and will add to the existing pool of knowledge.

## Materials and methods

This descriptive cross-sectional study was based in the department of medicine at the Military Hospital, Rawalpindi, Pakistan from December 2015 to April 2016. A sample size of hundred patients was obtained with the help of the World Health Organization (WHO) sample size calculator, (confidence interval CI = 95%, significance level = five percent, pooled standard deviation (SD) = 2.46). Patients aged between 12 and 65 years, diagnosed with essential hypertension of one year or more according to the Joint National Committee (JNC 7) guidelines were enrolled in the study. Patients with hematological system disorder, hepatic disease, renal disease, any systemic disease including rheumatologic disorders that could affect blood count, chronic hepatobiliary disease, peripheral artery disease, stroke, inflammatory bowel disease or diseases of terminal ileum malignant disorder and diabetes mellitus type 2 were excluded from the study. The study was approved by the hospital ethics committee and the institutional review board of the Military Hospital, Rawalpindi. Every participant was given 15 minutes to fill the proforma pertaining to the relevant clinical details. Informed consent of willing patients were taken and they were assured that their identity will be kept anonymous. The characteristics of the patients including age, gender, duration of hypertension and other medical conditions were recorded on a standardized data collection form. Blood samples were drawn and the complete blood count was determined by KX 21 Sysmex Auto Hematology Analyzer (Sysmex, Japan). The results were verified by the Armed Forces Institute of Pathology.

Data analysis was performed using SPSS Statistics V22.0 (IBM Corp., NY, USA). Mean and standard deviation (±SD) was calculated for all the quantitative variables, i.e. age, duration of hypertension, and serum RDW. The qualitative variables used were gender and hypertension. The normality of the study variables was tested with the Kolmogorov-Smirnov test. The independent sample t-test was applied to assess if there was any significant difference between age and gender. The Mann-Whitney test was used to assess any association of RDW with gender. Linear regression was done to assess if there was any correlation between age and RDW.

## Results

Out of 100 patients 69% (69/100) were males whereas 31% (31/100) were females. The mean age ± standard deviation (SD) of the patients was 51.48 ± 10.08 years. This is shown in Table 1. The overall frequency of hypertension more than five years was 55% (55/100) subjects whereas 45% (45/100) individuals had duration of hypertension less than five years. The mean RDW (± SD) was found to be 46.20 ± 7.79 fl with cut-off value taken less than 42.5 fl. The overall frequency of increased RDW was 71% (71/100 subjects) whereas 29% (29/100 individuals) had normal serum RDW. This is shown in Table 2. The mean RDW in females was found to be 49.35±8.42 fl while the mean RDW in males was 44.78±7.11 fl. The independent sample t-test was applied to assess if there was any significant difference between age and gender.

**Table 1:** Duration of Hypertension with Raised Red Cell Distribution Width (RDW)

Age and Duration of Hypertension	Frequency/ Percentage (N=100)	Mean ±Standard Deviation
Mean Age (Years)	-	51.4±10.08
Duration of Hypertension (≤5 Years)	45	-
Duration of Hypertension (≤5 Years)	55	-

**Table 2:** Frequency of Normal and Raised Red Cell Distribution Width (RDW)

Red Cell Distribution Width (N=100)	Frequency/Percentage	Mean±Standard Deviation
Mean RDW (femtoliters)	-	46.2±7.7
Normal RDW≤42.5 femtoliter	29/100 (29%)	-
Raised RDW≥42.5 femtoliters	71/100 (71%)	-

No significant difference between age and gender was found (p<0.05). The Mann-Whitney test was used to assess any association of RDW with gender. The RDW value in females was found to be statistically significantly higher than in males (U=603, p=0.01). Linear regression showed that the mean RDW value increased with increasing age (P <0.001).

## Discussion

Hypertension is currently the leading risk factor resulting in considerable death and disability worldwide, and it accounted for 9.4 million deaths and seven percent of disability adjusted life years (DALYs) in 2010 [20]. Risks of chronic kidney diseases and concomitant cardiovascular diseases make hypertension a serious health problem. The damage to the blood vessels due to damage of vascular endothelium in hypertension can lead to complications in other parts of the body [21]. RDW, a part of the normal complete blood count, is a measure of the variation in the size of circulating erythrocytes and is measured by automated hematology analyzers. RDW has been shown to be raised in various diseases that show inflammatory stress including prehypertension and hypertension [21]. It has been well documented in various studies that RDW can be used as a novel predictor of mortality in diseases such as coronary heart disease (CHD), stroke, peripheral artery disease (PAD), heart failure (HF), venous thromboembolism (VTE), and pulmonary arterial hypertension (PAH) [22].

Hypertension is a well-known risk factor for diseases like stroke, cardiovascular disease (CVD) and renal failure [22]. RDW is found to be increased in prehypertension and hypertension [4].Chronic inflammation may cause RDW elevation, and increased RDW levels might reflect an underlying chronic inflammation, which would explain the relationship between raised RDW in CVD. All these underlying causes result in an increased risk of CVD [23]. Our findings resemble that of Wen, et al. who found that there was a close relationship between RDW levels and carotid artery atherosclerosis in patients with hypertension [24]. Another community-based prospective cohort study conducted by Perlstein TS, et al. reported that increased RDW levels were associated with higher blood pressure levels [25]. Tonelli M, et al. found that higher levels of RDW were independently related to increased risk of death and cardiovascular events in people with hypertension [6]. Jithesh, et al. observed that inflammatory markers like high-sensitive C reactive protein (hs-CRP) and RDW levels were higher in the hypertensive patients compared to healthy participants [25]. Tanindi, et al. mentioned possible mechanisms by which RDW is increased in patients with high blood pressure. The first was activation of the renin angiotensin aldosterone system, in which angiotensin II leads to an increased erythropoietin production and early proliferation of erythroid progenitors. The second mechanism proposed was activation of adrenergic activity, leading to an increased sympathetic nervous system activity, which led to increased erythropoietin production [4]. Similarly Dan Su, et al. showed increased RDW values in hypertensives (all dipping patterns) (Table 3) [26].

**Table 3:** Red Cell Distribution Width (RDW) With Hypertension in View of Various Studies

Other Studies	Mean RDW ±Standard Deviation	P value
Ozcan F, et al. [3].	Non-dippers had significantly higher RDW levels than dippers [14.6 (13.8-17.0) vs 13.0 (12.5-13.4) respectively	<0.001
Tanindi A, et al. [6].	Hypertensives had higher 16.54 ± 0.91 RDW values than prehypertensives 15.26 ± 0.82 and controls 3.87 ± 0.94	<0.05
Dan Su, et al. [26].	Hypertensives (reverse dippers) had higher 13.52±1.05 RDW than hypertensives (dippers) 13.25±0.85	=0.012

In our study, the mean RDW value increased with increasing age. Researches have shown that inflammatory markers increase with increasing age [27]. This might contribute to increased anisocytosis with aging. Another reason for this difference can be the fact that Vitamin B-12 and folic acid deficiency are more common in the elderly as compared to younger patients. A study done by Rafael Alis, et al. had a similar conclusion [28]. In our study, females were found to have a statistically significant higher RDW value as compared to male participants. Rafael Alis, et al. and Lippi, et al. [29] had a similar conclusion. Women are more likely to have folic acid deficiency as compared to males and this might result in an increased RDW in female participants. Menorrhagia is one of the most common complaints of females of reproductive age. Menorrhagia has been associated with increased RDW [30]. RDW is widely available to physicians as part of the complete blood count and therefore does not increase additional costs, in contrast to other novel markers of cardiovascular risk.

The main limitations of our study were that we used a cross-sectional design and it was carried out in a single hospital setting; so results cannot be generalized. Hypertensive patients were not followed-up in terms of future adverse cardiovascular events. Although we demonstrated a significant association between elevated RDW in hypertensive patients, we did not investigate the specific markers of oxidative stress and inflammation to determine the exact mechanism of this association. We did not compare the RDW levels in the normal patients with same demographic characteristics.

## Conclusions

Our study shows RDW to be directly related with hypertension, thus giving weight to the hypothesis that RDW and inflammation are directly linked and that chronic inflammation can lead to an increase in RDW. Being a relatively easy and readily available test to perform, it can be used as an early warning system for physicians to identify prehypertension and hypertension in patients and to identify those patients who are at a greater risk for adverse outcomes from cardiovascular disease. Further studies with larger sample size are needed to shed light on the mechanism underlying this association.
